# Possible therapeutic effects of *Crocus sativus* stigma and its petal flavonoid, kaempferol, on respiratory disorders

**DOI:** 10.1080/13880209.2020.1844762

**Published:** 2020-12-09

**Authors:** Majid Kianmehr, Mohammad Reza Khazdair

**Affiliations:** a Esfarayen Faculty of Medical Sciences, Esfarayen, Iran; b Cardiovascular Diseases Research Center, Birjand University of Medical Sciences, Birjand, Iran

**Keywords:** Saffron, airway inflammation, smooth muscle relaxant effect

## Abstract

**Context:**

*Crocus sativus* L. (Iridaceae), or saffron, has been used as food additives and spices. In the traditional medicine of Iran, *C. sativus* has been used for the treatment of liver disorders, coughs, and as an anti-inflammatory agent for eyes.

**Objective:**

The current study reviewed the possible therapeutic effects of *C. sativus* stigma and its petal flavonoid (kaempferol) on respiratory disorders with several mechanisms such as anti-inflammatory, and smooth muscle relaxant effects.

**Materials and methods:**

This review article searched databases including PubMed, Google Scholar, and ScienceDirect, up to November 2019. The keywords including; ‘*Crocus sativus*’, ‘saffron’, ‘kaempferol’, ‘airway inflammation’, and ‘smooth muscle relaxant’ were searched.

**Results:**

*C. sativus* reduced nitric oxide (NO), inducible nitric oxide synthase (iNOS) levels and inflammatory cytokines in the lung tissue. Saffron and kaempferol reduced white blood cells (WBCs) and the percentage of neutrophils and eosinophils in bronchoalveolar lavage fluid. Moreover, saffron reduced tracheal responsiveness to methacholine and ovalbumin on tracheal smooth muscles. In addition, kaempferol reduced the total leukocyte and eosinophil counts similar to the effect of dexamethasone and also showed relaxant effects on smooth muscle.

**Discussion and conclusion:**

*Crocus sativus* and its petal flavonoid, kaempferol, showed relatively potent therapeutic effects on respiratory disorders by relaxation of tracheal smooth muscles via stimulatory or blocking effects on β-adrenoceptor and muscarinic receptors, respectively. Saffron and kaempferol also decreased production of NO, inflammatory cytokines and chemokines in respiratory systems.

## Introduction

Asthma is a complex inflammatory disorder characterised by airway inflammation and hyperresponsiveness, hypersecretion of mucus by goblet cells and eosinophilic inflammation (Bousquet et al. 2000). Asthma is triggered by a very complex interaction between high serum levels of immunoglobulin E (IgE) and production of inflammatory mediators such as; interleukin (IL)-4, IL-5, and IL-13 by T-helper 2 (Th2) cells (Anderson and Coyle 1994). Asthma is a two-module disease including airway inflammation (Hoogsteden et al. 1999) and smooth muscle dysfunction (Janssen and Killian 2006). Chronic obstructive pulmonary disease (COPD) is a type of obstructive lung disease that is characterised by airway remodelling and inflammation, mucus hypersecretion, and emphysema, which leads to reduction in lung function and breathlessness (Barnes 2014). It has been reported that lung diffusion capacity was declined, while respiratory symptoms, lung hyperinflation and induced-sputum neutrophil and bronchial cell counts were increased in COPD patients (Boulet et al. 2006).

Inflammation is enhanced by local responses of the epithelium, fibroblasts cells and smooth muscle, through the production of cytokines, and proteases. The main characteristic of asthma is increased airway responsiveness which is due to lung inflammation (Cohn et al. 2004).

Corticosteriods are capable of inhibiting eosinophil function or infiltration and expression of multiple inflammatory genes such as, cytokines, enzymes, receptors and adhesion molecules, and also bronchodilatory drugs are anticipated for the treatment of asthma (Sears et al. 1990; Barnes 2006).

It has been reported that combination therapy with corticosteroids and β-agonists reduced tracheal hyperresponsiveness and lung inflammation in an ovalbumin (OVA)-sensitised animal model (Khazdair et al. 2013; Gholamnezhad et al. 2014). Medicinal plants have long been used in traditional medicine for the treatment of various inflammatory disorders such as asthma. Medicinal plants used for asthma should have anti-inflammatory, antihistaminic, immunomodulatory and smooth-muscle relaxants activity (Greenberger 2003; Khazdair et al. 2019b).

*Crocus sativus* L. (Iridaceae), or saffron, is commonly cultivated in Iran, Afghanistan, Turkey and Spain (Khazdair et al. 2015). *C. sativus* has been used for the treatment of liver disorders, coughs, and as an anti-inflammatory agent in Iranian traditional medicine (Abrishami 1987). *C. sativus* and its constituents have been shown to have beneficial effects on the coronary artery (Xu et al. 2005), respiratory (Boskabady and Aslani 2006; Boskabady et al. 2010; Mokhtari-Zaer et al. 2015), nervous system (Mohebbati et al. 2017; Khazdair et al. 2019a) and gastrointestinal diseases (El-Maraghy et al. 2015). The effects of saffron and its constituents on respiratory disorders in traditional medicine were reviewed (Boskabady et al. 2019).

Kaempferol is a flavonoid that can be extracted in good quantities from the petals of the *C. sativa* (Zeka and Arroo 2016). The wide range of pharmacological properties for kaempferol, including antioxidant, anticancer, and anti‐inflammatory effects, have been reported (Imran et al. 2019). The anti-inflammatory and smooth muscle relaxant effects (anti-asthmatic effects) of a flavonoid have been reported (Khazdair et al. 2019b).

The current study reviewed the potential therapeutic effects of *C. sativus* stigma and its petal flavonoid (kaempferol) with several mechanisms, such as anti-inflammatory and smooth muscle relaxant effects on inflammatory respiratory diseases.

## Methods

All literature that reported the effects of *Crocus sativus* stigma and its petal flavonoid, kaempferol on respiratory disorders were selected in PubMed, Google Scholar, and ScienceDirect, up to November 2019. The keywords including; ‘*Crocus sativus*’, ‘saffron’, ‘Kaempferol’, ‘airway inflammation’, and ‘smooth muscle relaxant’ were searched individually or combined.

## Results

### Anti-inflammatory effects of saffron

#### *In vitro* studies

The macerated extracts of *C. sativus* (50, 250, and 500 μg/mL) on cell viability and cytokine release of stimulated peripheral blood mononuclear cells (PBMC) by phytohemagglutinin (PHA) and non-stimulated cells significantly inhibited cell viability of lymphocytes and secretion of interferon-γ (IFN-γ) in stimulated cells and also inhibited interleukin (IL)-10 secretion in stimulated and non-stimulated cells (Boskabady et al. 2011). Treatment HepG2 cells (*in vitro*) with crocin (0.01, 0.03, 0.1, 0.3, 1 mM) for 24 and 48 h reduced cell viability at dose dependently. Crocin also significantly decreased IL-8 secretion and protein levels of TNF receptor 1 (TNFR1) by HepG2 cells at 6 and 12 h after treatment (Amin et al. 2016).

Treatment of normal human bronchial epithelial cells with methanol water extract of *C. sativus* (10 and 100 ng/mL) and its constituents safranal resulted in a decrease of nitric oxide (NO), inducible nitric oxide synthase (iNOS) levels, peroxynitrite ion generation, and prevented cytochrome c release (Bukhari et al. 2015).

#### *In vivo* studies

Oral administration of *C. sativus* extract (1 and 10 mg/kg, p.o.) and safranal in the OVA-induced murine model of asthma (*in vitro*) study, reduced iNOS levels and inflammatory cytokines such as; L-5 and IL-13 levels in the lung tissue. Airway hyper‐responsiveness and airway cellular infiltration to the lungs as well as, bronchial epithelial cell apoptosis was decreased in treated mice with *C. sativus* and safranal (Bukhari et al. 2015). Oral administration of crocin (100 and 200 mg/kg b.w.) significantly decreased elevated levels of cyclooxygenase-2 (COX-2) and iNOS in diethylnitrosamine (DEN) induced hepatocellular carcinoma in male albino Wistar rats (Amin et al. 2016).

Hydroethanol extract of saffron in drink water (0.1, 0.2 and 0.4 mg/mL) on OVA-sensitised induced asthma in guinea pigs significantly reduced serum levels of endotheline-1 (ET-1) and total protein (TP) compared to untreated OVA-sensitised guinea pigs (Gholamnezhad et al. 2013). Treatment of sensitised animals with *C. sativus* extract and dexamethasone reduced serum levels of ET-1 and TP. Furthermore, the extract of the plant (0.4 mg/mL) was higher effective than dexamethasone (50 µg/mL) (Gholamnezhad et al. 2013).

The effects of *C. sativus* extract and dexamethasone treatment on lung inflammation in an animal model of allergic asthma showed the hydroethanol extract of saffron (0.1, 0.2 and 0.4 mg/mL) prevented the increase in total white blood cells (WBC), eosinophil and lymphocyte numbers in OVA-sensitised animals. The extract reduced WBC count similar to dexamethasone. In addition, the low concentration of extract (0.1 mg/mL) reduced eosinophil count more than dexamethasone treatment (Bayrami and Boskabady 2012; Boskabady et al. 2012). Treatment of sensitised animals with the plant extract significantly ameliorated lung pathological indices. These data also suggest a preventive effect of saffron extract on lung inflammation of sensitised guinea pigs (Boskabady et al. 2012). Treatment of OVA-sensitised rats with hydroethanol extract of saffron (50, 100 and 200 mg/kg) reduced WBC number and decreased the percentage of neutrophils and eosinophils in bronchoalveolar lavage fluid (BALF) as compared to the non-treated sensitised animals (Mahmoudabady et al. 2013). In a similar study *C. sativus* hydroethanol extract (50, 100, and 200 mg/kg) also significantly decreased WBC count, eosinophil percentage, neutrophil percentage, red blood cell (RBC) and platelet count in the blood of sensitised rats. Lymphocyte percentage was increased in animals receiving 100 mg/kg of saffron hydroethanol extract (Vosooghi et al. 2013).

Administration of *C. sativus* hydroethanol extract (20, 40, and 80 mg/kg/day) remarkably reduced tracheal responsiveness, as main characteristic of obstructive respiratory diseases especially asthma, to both methacholine and OVA and also decreased serum levels of inflammatory mediators compared to untreated sensitised animals. In addition, *C. sativus* extract (80 mg/kg) was more effective than those of dexamethasone (10 mg/kg). These findings indicated that the extract of the plant could attenuate serum levels of inflammatory mediators as well as increase tracheal responsiveness to methacholine and OVA (Byrami et al. 2013).

The results of these studies indicated the preventive effect of *C. sativus* on reduction of tracheal responsiveness, total proteins and airway inflammation may indicate the therapeutic effect of the plant on allergic asthma. The reduction of eosinophil, neutrophil and lymphocyte count in sensitised animals treated with *C. sativus* extract suggests that saffron has anti-inflammatory activity. In addition, decreased RBC and platelet counts as well as inflammatory cytokines in sensitised animals treated with saffron extract indicated that the plant extract may be a useful treatment for different inflammatory lung diseases.

Intrapritoneal administration of ethanol and aqueous extracts of *C. sativus* (200 mg/kg, i.p.) reduced neuropathic pain in the chronic constriction injury (CCI) model through attenuation levels of pro-inflammatory factors including: tumour necrosis factor α (TNF-α), IL-1 β and IL-6 , on the lumbar spinal cord (Amin et al. 2014). Aqueous (0.8 g/kg; i.p.) and ethanol extracts of *C. sativus* (0.1, 0.2 and 0.4 g/kg, i.p.) intraperitoneal administrated in mice showed antinociceptive activity against acetic acid-induced writhing. Also, the plant extracts showed mild to moderate effects against acute inflammation caused by xylene in mice ear edoema. In addition, both aqueous and ethanol *C. sativus* extracts, showed anti-inflammatory effects by formalin-induced edoema in rat paw in induced chronic inflammation (Hosseinzadeh and Younesi 2002). The results of this study indicated that aqueous and ethanol extracts of saffron in addition to acute and/or chronic anti-inflammatory activity have antinociceptive effects.

Oral administration of ethanol extract of saffron (200 mg/kg, p.o.) in the treatment of experimental autoimmune encephalomyelitis (EAE) in C57BL/6 mice significantly reduced the clinical symptoms in C57BL/6 mice with EAE. Also, treated mice displayed a delayed disease onset compared with control mice. Antioxidant capacity production was significantly elevated in saffron treated mice. Furthermore, mice treated with saffron reduced typical spinal cord leukocyte infiltration compared with control mice. These results suggested that saffron is effective in the prevention of symptomatic EAE by inhibition of oxidative stress and leukocyte infiltration to the central naevus system and may be potentially useful for the treatment of multiple sclerosis (MS) (Ghazavi et al. 2009).

White blood cells (WBC) were significantly increased in patients receiving the *C. sativus* capsule (15 mg) twice daily compared with patients receiving a placebo in schizophrenia in which WBC count was in the normal range. Moreover, the other haematologic components and markers did not change significantly during 3 months of the study (Mousavi et al. 2015).

Treated monocytes (1 × 106 cells/mL) of both healthy donors and Alzheimer’s disease (AD) subject with a constituent of *C. sativus,* *trans*-crocetin (5, 10, 25, 50, 100, and 150 μM) enhanced amyloid-β 42 (Aβ42) degradation in AD monocytes through the up-regulation of the lysosomal protease cathepsin B (Tiribuzi et al. 2017).

These results indicated that saffron and its components may be useful in treating CNS disorders by reducing oxidative stress and reducing inflammation. Anti-inflammatory effects of saffron is summarised in Table 1.

**Table 1.** Anti-inflammatory effects of saffron extracts.

Extract	Effect	Experimental model	Ref.
Macerated extracts	Inhibited cell viability of lymphocytes and secretion of IFN-γ	Peripheral blood mononuclear cells	Boskabady et al. (2011)
Methanol extract	Decreased NO, and iNOS levels, and also prevented cytochrome c releases	Human bronchial epithelial cells	Bukhari et al. (2015)
Aqueous extract	Reduced iNOS levels and inflammatory cytokines such as; L-5 and IL-13 levels in the lung tissue. airway hyper‐responsiveness and airway cellular infiltration to the lungs	Murine model of asthma	Bukhari et al. (2015)
Hydroethanolic extract	Reduced serum levels of endotheline-1 (ET-1) and total protein (TP)	Sensitised guinea pigs	Gholamnezhad et al. (2013)
	Reduced total WBC, eosinophil and lymphocyte counts in blood and lung lavage		Bayrami and Boskabady (2012)
	Ameliorated lung pathological indices		Boskabady et al. (2012)
	Decreased tracheal responsiveness to both methacholine and OVA and serum levels of inflammatory mediators		Byrami et al. (2013)
	Reduced WBC number and decreased the percentage of neutrophils and eosinophils in lung lavage	Sensitised rats	Mahmoudabady et al. (2013)
	Reduced WBC, RBC and platelet count, Reduced Eosinophil and neutrophil percentage. Increased Lymphocyte percentage		Vosooghi et al. (2013)
Ethanolic extract	Attenuation of pro-inflammatory factors (TNF-α, IL-6 and IL-1β)	Chronic constriction injury in rat	Amin et al. (2014)
Ethanolic extract	Reduced ear edoema and showed antinociceptive effects	Acetic acid-induced writhing in mice	Hosseinzadeh and Younesi (2002)
Aqueous extract of petal	Ameliorated paw edoema	Formalin-induced paw edoema	Hosseinzadeh and Younesi (2002)
Ethanolic extract	Delayed disease onset, Elevated antioxidant Capacity and reduced leukocyte infiltration to CNS	Mice model of autoimmune encephalomyelitis	Ghazavi et al. (2009)
*C. sativus* capsule	Increased WBC	Schizophrenia patients	Mousavi et al. (2015)
Constituent of *C. sativus*	enhanced Amyloid-β 42 (Aβ42) degradation in Alzheimer's Disease monocytes	Human monocytes	Tiribuzi et al. (2017)

### Anti-inflammatory effects of kaempferol

#### *In vitro* studies

Kaempferol is a polyphenolic compound isolated from the fresh flower petals of saffron (Hadizadeh et al. 2010). The effects of kaempferol on epithelial-to-mesenchymal transition (EMT) and cell migration induced by transforming growth factor-β1 (TGF-β1) in human non-small lung cancer cells (A549) showed that kaempferol (10, 25, and 50 µM) significantly blocked the increased cell migration by TGF-β1 induced EMT through recovering the loss of E-cadherin and blocking the induction of mesenchymal markers as well as the upregulation of TGF-β1 mediated matrix metalloproteinase-2 (MMP-2) activity. Furthermore, activation of kinase (Akt1) was required for TGF-β1 mediated induction of EMT and cell migration and directly phosphorylated a protein (Smad3) at Thr179, and kaempferol completely eliminated TGF-β1-induced Akt1 phosphorylation. These results indicated that kaempferol blocks TGF-β1 induced EMT and migration of lung cancer cells by inhibiting Akt1 which mediated phosphorylation of Smad3 at Thr179 residue (Jo et al. 2015).

Kaempferol (20, 40, 60, 80, and 100 μM) also suppressed mRNA expression of MMP-2 to restrain the migration of oral cancer cells via inhibiting the c-Jun pathway and extracellular signal-regulated protein kinases 1 and 2 (ERK1/2) phosphorylation in a dose-dependent manner (Lin et al. 2013). Furthermore, kaempferol (5–20 μM) significantly reduces vascular endothelial growth factor (VEGF) gene expression at mRNA and protein levels and significantly inhibited angiogenesis and tumour growth. Moreover, kaempferol treatment, down-regulated HIF-1α (a regulator of VEGF) dose dependently in ovarian cancer cell line (Luo et al. 2009).

Kaempferol (20 μM) inhibited secretion of β-hexosaminidase and histamine, and reduced the production and mRNA expression of inflammatory cytokines (IL-4 and TNF-α) in immunoglobulin E (IgE)-sensitised RBL-2H3 cells. Kaempferol also inhibited (IgE)-mediated phosphorylation of phospholipase Cγ, protein kinase C (PKC), and the mitogen-activated protein kinases, extracellular signal-regulated kinase, p38, and c-Jun N-terminal kinase (Kim et al. 2014). The effects of kaempferol on the IL-1β induced proliferation of rheumatoid arthritis synovial fibroblasts (RASFs) and the production of MMPs, cyclooxygenase (COX)-2 and prostaglandin E2 (PGE2) showed that kaempferol (100 μM) inhibited the proliferation of unstimulated and IL-1β stimulated RASFs in addition to the mRNA and protein expression of MMP-1, MMP-3, COX-2 and PGE2 induced by IL-1β. Kaempferol inhibited the activation of nuclear factor-kappa B (NF-κB) induced by IL-1β and also inhibited the phosphorylation of ERK-1/2, p38 and JNK (Yoon et al. 2013). Kaempferol (30 μM) significantly decreased the mRNA expression of TNF-α in LPS-activated J774.2 macrophages. IL-1β gene expression in LPS-induced J774.2 macrophages also inhibited by kaempferol (Kowalski et al. 2005). LPS (100 ng/mL) enhanced iNOS mRNA expression, NF-κB activity and signal transducer, and activator of transcription 1 (STAT-1), which are important transcription factors for iNOS in which kaempferol (10–100 μM) considerably inhibited iNOS protein and mRNA expression as well as NO production. Kaempferol also inhibited the activation of NF-κB and STAT-1 in a dose-dependent manner in J774 macrophages (Hämäläinen et al. 2007). Treatment of diabetic mice with kaempferol (25, 50 and 100 mg/kg, p.o) attenuated the development of diabetic neuropathy and reduced pain sensation. Furthermore, kaempferol reduced IL-1β, TNF-α, lipid peroxidation and nitrite (Abo-Salem 2014).

It was demonstrated that kaempferol (30 and 150 mg/kg, p.o.) decreased the levels of TNF-ɑ and IL-1β in serum of high cholesterol-fed rabbits. In addition, kaempferol respectively down-regulated mRNA and protein expression of inflammatory molecules such as E-selectin (E-sel), intercellular adhesion molecule-1 (ICAM-1), vascular cell adhesion molecule-1 (VCAM-1) and MCP-1 in the aorta of rabbits (Kong et al. 2013).

The effects of kaempferol (1–20 µM) on inflammation in human airway epithelial cells (BEAS-2B) showed kaempferol inhibited the expression of Toll-like receptor 4 (TLR4) a promotor of inflammatory mechanisms which significantly increased by LPS. Kaempferol also decreased the cellular expression of IL-8 through stimulating (Gong et al. 2013).

Kaempferol (10-20 μM) inhibited mast cell degranulation and prostaglandin release, leading to the development of aberrant airways in basophilic leukaemia (RBL-2H3) mast cells obtained from dinitrophenylated bovine serum albumin (DNP-BSA)-sensitised rat. Kaempferol suppressed β-hexosaminidase release and COX-2-mediated production of prostaglandin D2 (PGD2) and prostaglandin F2α (PGF2α) in sensitised mast cells. Kaempferol prevented the antigen-induced mast cell activation of cytosolic phospholipase A2 (cPLA2) responsive to protein kinase Cμ (PKCμ) and extracellular signal-regulated kinase (ERK). These results demonstrated that kaempferol inhibited ERK-cPLA2-COX-2 signalling in mast cells (Shin et al. 2015).

Kaempferol (1–20 μM) significantly decreased LPS-induced cellular levels of transforming growth factor beta 1 (TGF-β1) in a dose-dependent manner in BEAS-2B cells. Furthermore, LPS stimulation significantly induced TGF-β RI and TGF-β RII, which was reversed by kaempferol. Epithelial E-cadherin expression was substantially increased by kaempferol, which was dampened by TGF-β. Kaempferol (1–20 μM) suppressed the epithelial production of collagen IV, which was enhanced by LPS in BEAS-2B cells. Thus, kaempferol may improve epithelial barrier function by cell-cell adhesion (Gong et al. 2014).

The antioxidant and anti-inflammatory effects of kaempferol including; inhibitory effects on COX-1 and 2 enzymes and mitogen-activated protein kinase (MAPK) pathway in human monocytic cell line THP-1 were reviewed (Devi et al. 2015). Administration of kaempferol (0, 1, 2 or 4 μg/mL) suppressed expression of the major inflammatory cytokines TNF-α, IL-6, IL-1β and PGE2 in cultured RAW macrophages. Kaempferol also decreased oxidative stress in cultured cells (Sun et al. 2019).

The results of the *in vitro* studies indicated that kaempferol has anti-inflammatory properties via inhibition iNOS protein, prostaglandin and NO production as well as mRNA expression. This flavonoid also inhibited the activation of NF-κB and STAT-1 in macrophages, and reduced inflammatory cytokines including; TNF-α, IL-6, IL-1β and PGE2 and also increased inflammatory cytokines *in vitro*, which it can useful for the treatment of allergic disorders such as asthma.

#### *In vivo* studies

Intra-gastric treatment with kaempferol (100 mg/kg, i.g) on lipopolysaccharide (LPS)-induced lung injury in BALB/c mice strongly reduced overproduction of pro-inflammatory cytokines in BALF, including TNF-α, IL-1β and IL-6. Kaempferol also significantly inhibited LPS-induced alveolar wall thickness, leukocytes infiltration and alveolar haemorrhage in lung tissue with evidence of reduced myeloperoxidase (MPO) activity and increased superoxide dismutase (SOD) activity. Furthermore, kaempferol significantly blocked the activation of mitogen-activated protein kinases (MAPKs) and NF-κB signalling pathways induced by LPS. These results suggested that kaempferol exhibits a protective effect on LPS-induced acute lung injury by suppressing MAPKs and NF-κB signalling pathways, involved the inhibition of oxidative injury and inflammatory process (Chen et al. 2012).

The levels of C–C chemokine receptor type 3 (CCR3) and eotaxin-1 protein in the lung tissue were enhanced in OVA-exposed mice, but the supplementation of kaempferol (10 and 20 mg/kg, p.o) dose-dependently eliminated the production levels of CCR3 and eotaxin-1. OVA challenge also increased macrophage inflammatory protein 2 (MIP-2) and C-X-C chemokine receptor type 2 (CXCR2) production in mouse lung tissue which kaempferol supplemented markedly reduced MIP-2 and CXCR2 production (Gong et al. 2013).

Kaempferol could be capable of modulating allergic airway disease (AAD) either as a preventive (administered 1 h before OVA-sensitisation) or curative (OVA-sensitisation at day 18–21) treatment in sensitised mouse models (Medeiros et al. 2009). Kaempferol (3, 30 or 100 mg/kg, s.c) also reduced the total leukocyte and eosinophil counts similar to the effect of dexamethasone (1 mg/kg) in the BALF (Medeiros et al. 2009).

Kaempferol (50 mg/kg, p.o.) markedly inhibited the passive cutaneous antigen-induced anaphylaxis (PCA) response in IgE-sensitised mice (Kim et al. 2014). It has been reported that kaempferol (2 or 4 mg/kg/day, p.o.) can inhibit NF-κB function by inhibiting the activation of nuclear factor-inducing kinase (NIK)/I*κ*B kinase (IKK) and MAPKs signal pathways in aged rat kidney (Park et al. 2009). Therefore, kaempferol plays anti-inflammatory roles by modulating the gene and protein expression of inflammatory molecules.

Oral administration of kaempferol (10 and 20 mg/kg) suppressed bovine serum albumin inhalation-induced epithelial cell excrescence and hypertrophy in smooth muscle by attenuating the induced COX-2 and the formation of PGD2 and PGF2α, as well as reduced the expression of anti-α-smooth muscle actin in mouse airways. These results demonstrated that kaempferol inhibited airway wall thickening through disturbing Syk-PLCγ signalling and suggested it may be a potent anti-allergic compound that targeting of allergic asthma (Shin et al. 2015).

Oral administration of kaempferol (10 or 20 mg/kg) to OVA-challenged mice induced pulmonary TGF-β1 induction reduced TGF-β RI and levels of the epithelial marker E-cadherin in lung tissues of OVA-challenged mice. In addition, kaempferol (10 or 20 mg/kg) significantly reduced the lung tissue level of fibrogenic collagen IV which increased in mice sensitised with 5% OVA (Gong et al. 2014).

Administration of kaempferol (10 and 20 mg/kg) reduces omega-6 and ovalbumin-induced allergic reactions at lung and trachea in BALB/c mice. It also inhibited the increased histamine level and expression level of COX-2 which increased in the lung and trachea after OVA sensitisation. The results of this study suggested kaempferol might have a positive effect in mitigating allergic inflammatory response of the respiratory tract (Belal et al. 2018).

Orally administered of kaempferol (100 mg/kg) 1 h before caecal ligation and puncture surgery in mice significantly decreased water content in the lungs. Pre-treatment with kaempferol also reduced cytokines, such as IL-6, IL-1β, and TNF-α in the plasma and in the lung tissue compared to the untreated mice. Kaempferol increased SOD and catalase and non-enzymatic antioxidant glutathione (GSH) activities in septic mice. Additionally, kaempferol reduced the lung tissue nitrite level and iNOS level and down regulated mRNA expression of intercellular adhesion molecule 1 (ICAM-1) and iNOS in septic mice (Rabha et al. 2018). Intraperitoneal administration of kaempferol (50 mg/kg) 30 min before challenging the mice with LPS reduced LPS-mediated production of cytokines including; IL-1β, TNF-α, IL-6. Kaempferol also reduced activation of NF-κB, iNOS, and COX-2, in lung tissues of mice (Qian et al. 2019). Intraperitoneal administration of kaempferol (50–200 μg/kg) significantly reduced the level of inflammatory cytokines including; TNF-α, IL-6, IL-1β and PGE2 in lung lavage fluid as dose-dependent manner and ameliorated lung edoema in Balb/c mice. In addition, the administration of kaempferol also significantly decreased MPO and malondialdehyde (MDA) and elevated SOD and GSH compared to control mice (Sun et al. 2019).

A recent study provided evidence that the flavonoids, reduced airway inflammation, inflammatory cells infiltration, Th2 cytokines, and allergen-specific IgE in a murine model of asthma. Mast cells play a critical role in the induction and progression of asthma due to release mediators that promote the interaction with distinctive cell types in asthma (Komi and Bjermer 2019). It has been reported that the flavonoids have potent antioxidant properties to scavenge free radicals and decreased their formation. Flavonoids have a deep impact on several immune cells and immune mechanisms that are important in the inflammatory processes and showed inhibitory effects on the release of mediators that implicated in asthma (Maleki et al. 2019).

The results of the above studies indicated that kaempferol reduced the overproduction of pro-inflammatory cytokines in BAL fluid of induced lung injuries in animal models. Kaempferol also inhibited induced alveolar wall thickness, leukocytes infiltration and alveolar haemorrhage in lung tissue. This flavonoid also has protective effects on stress oxidative via reduced myeloperoxidase (MPO) activity and increased superoxide dismutase (SOD) activity. Kaempferol also reduced the total leukocyte and eosinophil counts similar to dexamethasone used for the treatment of allergic inflammatory diseases. The anti-inflammatory effects of kaempferol are summarised in Table 2.

**Table 2.** Anti-inflammatory effects of kaempferol.

Effect	Experimental model	Ref.
Blocked the cell migration, upregulation of MMP-2 activity and eliminated TGF-β1 induced Akt1 phosphorylation.	Lung cancer cells	Jo et al. (2015)
Suppressed mRNA expression of MMP-2	Oral cancer cells	Lin et al. (2013)
Reduces VEGF gene expression at mRNA and protein levels and significantly inhibited angiogenesis and down regulated of HIF-1α	Ovarian cancer cell	Luo et al. (2009)
Inhibited secretion of β-hexosaminidase and histamine, and reduced the production of inflammatory cytokines	RBL-2H3 cells	Kim et al. (2014)
Kaempferol inhibited the activation of NF-κB and phosphorylation of ERK-1/2, p38 and JNK	RASFs cells	Yoon et al. (2013)
Reduced mRNA expression of TNF-α and inhibition of IL-1β gene expression	LPS-activated J774.2 macrophages	Kowalski et al. (2005)
Inhibited iNOS protein and mRNA expression as well as NO production, and inhibited the activation of NF-κB and STAT-1	J774 macrophages	Hämäläinen et al. (2007)
Significantly decreased LPS-induced cellular levels of TGF-β1. Furthermore, LPS stimulation significantly by kaempferol. Epithelial E-cadherin expression was substantially increased by kaempferol, which dampened by TGF-*β*. Kaempferol suppressed the epithelial production of collagen IV, and reversed induced TGF-*β* RI and TGF-*β* RII, which was enhanced by LPS.	BEAS-2B cells	Gong et al. (2014)
Suppressed expression of the major inflammatory cytokines TNF-α, IL-6, IL-1β and PGE2 and also decreased oxidative stress in cultured cells	RAW macrophages	Sun et al. (2019)
Attenuated the development of diabetic neuropathy and reduced pain sensation and reduced IL-1β, TNF-α, lipid peroxidation and nitrite	Diabetic mice	Abo-Salem (2014)
Down-regulated mRNA and protein expression of E-selectin, ICAM-1, VCAM-1 and MCP-1	High cholesterol rabbits	Kong et al. (2013)
Inhibited the expression of TLR4 and decreased cellular expression of IL-8	BEAS-2B cells	Gong et al. (2013)
Inhibited ERK-cPLA2-COX2 signalling	(DNP-BSA) -sensitised rat	Shin et al. (2015)
Reduced TNF-α, IL-1β and IL-6, inhibited wall thickness, leukocytes infiltration and alveolar haemorrhage in lung tissue. In addition, significantly blocked the activation of MAPKs and NF-κB signalling pathways	LPS-induced BALB/c mice	Chen et al. (2012)
Eliminated the levels of CCR3 and eotaxin-1 and reduced MIP-2 and CXCR2 production	OVA-exposed mice	Gong et al. (2013)
Reduced TGF-*β* RI and levels of the epithelial marker E-cadherin in lung tissues of mice. In addition, kaempferol significantly reduced the lung tissue level of fibrogenic collagen IV which increased in mice sensitised with 5% OVA.	OVA-challenged mice	Gong et al. (2014)
Reduced the total leukocyte and eosinophil counts	OVA-sensitisation mice	Medeiros et al. (2009)
Inhibited the antigen-induced passive PCA	IgE-sensitised mice	Kim et al. (2014)
Inhibited NF-κB function, (NIK)/i*κ*b kinase (IKK) and MAPKs signal pathways	Aged rat	Park et al. (2009)
Reduced induced COX2, the formation of PGD2 and PGF2α and expression of anti-α-smooth muscle actin	Epithelial cell in mouse airways	Shin et al. (2015)
Increased histamine level and expression level of COX2	BALB/c mice	Belal et al. (2018)
Significantly decreased water content in lungs. Kaempferol also reduced cytokines such as, IL-6, IL-1β, and TNF-α in the plasma and in the lung tissue of mice. Kaempferol increased SOD and catalase and non-enzymatic antioxidant glutathione (GSH) activities in septic mice. Additionally, kaempferol reduced the lung tissue nitrite level and iNOS level and down regulated mRNA expression of intercellular adhesion molecule 1 (ICAM-1) and iNOS in septic mice.	Caecal ligation and puncture induced sepsis in mice	Rabha et al. (2018)
Administration of kaempferol 30 min before challenging the mice with LPS, mediated production of cytokines including; IL-1β, TNF-α, IL-6. Kaempferol also reduced activation of NF-κB, iNOS, and COX-2, in lung tissues of mice.	LPS-induced mice	Qian et al. (2019)
Significantly reduced the level of inflammatory cytokines including; TNF-α, IL-6, IL-1β and PGE2 in lung lavage fluid as dose-dependent manner and ameliorated lung edoema in Balb/c mice. In addition, administration of kaempferol also significantly decreased MPO and malondialdehyde (MDA) and elevated SOD and GSH compared to control mice.	*K. pneumoniae* infected mice	Sun et al. (2019)

#### The relaxant effect of *C. sativus* on smooth muscle

The aqueous ethanol extract of *C. sativus* (0.15, 0.3, 0.45, and 0.60 g %) showed a potent relaxant effect on guinea‐pig precontracted tracheal chains (*in vitro*) by methacholine (10 μM) when compared with saline treatment. Furthermore, there was a positive correlation between increasing concentrations of the extract and the relaxant effects (Boskabady and Aslani 2006).

Oral administration of hydro-ethanol extracts of *C. sativus* (20, 40, and 80 mg/kg/day) reduced tracheal responsiveness to methacholine in ovalbumin (OVA) sensitised guinea pigs (Byrami et al. 2013).

The antitussive effect of the intraperitoneal administration of ethanol extract of *C. sativus* (100–800 mg/kg) by reducing the number of cough was observed, which could be due to its relaxant effect on airway smooth muscle (Hosseinzadeh and Ghenaati 2006). The aqueous ethanol extracts from *C. sativus* (0.1 and 0.2%), and 10 nM propranalol (β-blockers) precontracted isolated guinea pig tracheal smooth muscle showed clear leftward shifts in isoprenaline (β adrenoreceptor agonist) curves obtained in the presence of saffron extract compared to that of saline, while propranolol caused rightward shift in isoprenaline response curve. This result indicated a potent stimulatory effect for *C. sativus* extract on β2-adrenoreceptors (Nemati et al. 2008). Similarly, the effect of *C. sativus* (25, 50 and 100 μg/mL) on muscarinic receptors of guinea pig tracheal chains showed the functional antagonistic effect of the extracts on muscarinic receptors on smooth muscle of tracheal chain (Neamati and Boskabady 2010). Moreover, the aqueous-ethanol extract of *C. sativus* (0.025, 0.05, and 0.1 mg/mL) on 10 nM chlorpheniramine, precontracted isolated guinea pig tracheal smooth muscle showed inhibitory effect of the plant extract on histamine H1 receptors (Boskabady et al. 2010).

Intravenous administration of aqueous extract of *C. sativus* (2.5, 5 and 10 mg/kg, i.v.), in normotensive and hypertensive rat model, reduced the mean arterial blood pressure and heart rate in a dose‐dependent manner. Additionally, administration of 10 mg/kg of saffron reduced the mean systolic blood pressure (MABP) by 60 ± 8.7 mmHg. These results suggested that *C. sativus* extract and its two active components have hypotensive properties in an animal model of hypertensive (Imenshahidi et al. 2010).

Aqueous and ethanol extracts of *C. sativus* petals reduced blood pressure in a dose-dependent manner in isolated rat vas deferens. Administration of 50 mg/g of aqueous extract reduced blood pressure 17 mmHg compared to the control group. This hypotensive effect could be either due to the effect of the *C. sativus* petals extracts on the heart itself or on total peripheral resistance via relaxation of vascular smooth muscle, or both. However, the results suggested that the effect of extracts on peripheral resistance seems to be the more probable mechanism of this effect (Fatehi et al. 2003).

It was also shown that the chronic administration of the aqueous extract of saffron (10, 20, and 40 mg/kg/day) in desoxycorticosterone acetate (DOCA) salt induced hypertensive in rats resulting in reduced MSBP in a dose-dependent manner, but this hypotensive effect was not observed in normotensive rats. Data showed that antihypertensive effects of saffron did not last long, so it could be postulated that long-term blood pressure regulation systems are not affected by saffron (Imenshahidi et al. 2013). Aqueous ethanol extract of *C. sativus* (0.1, 0.5, 1.0, and 5.0 mg%) showed a concentration-dependent inhibitory effect on heart rate and contractility, which was comparable to the effect of diltiazem. The effect of plant extract on heart contractility could be due to its muscle relaxant effect (Boskabady et al. 2008).

The aqueous extract of *C. sativus* (0.5, 1, and 2 mg/mL), on isolated rat thoracic aorta rings (tissue) were contracted by 10^−6^ M phenylephrine (PE) or KCl 80 mM showed vasodilatory effect on intact and denuded endothelium aortic rings. The extract induced relaxation in a dose-dependent manner in endothelium-intact aortic rings precontracted with PE. The relaxation effects of plant extract in precontracted tissue with KCl was less than that of precontracted tissue with PE. The relaxant activity of *C. sativus* was abolished by incubation of tissue with L-NAME but not in the presence of indomethacin. These results suggested *C. sativus* induced relaxation in isolated rat aortic tissue due to its effect on endothelium via nitric oxide synthase and the effect on vascular smooth muscle cells via L-type voltage-dependent calcium channels (Razavi et al. 2018). The relaxant effects of *C. sativus* is shown in Table 3.

**Table 3.** The relaxant effect of saffron on smooth muscle.

Extract	Effect	Experimental model	Ref.
Aqueous extract	Relaxant effect on guinea‐pig precontracted tracheal chains	Guinea‐pig tracheal chains	Boskabady and Aslani (2006)
Hydroethanolic extract	Reduced tracheal responsiveness to methacholine	OVA sensitised guinea pigs	Byrami et al. (2013)
Ethanolic extract	Relaxant effect on airway smooth muscle	Guinea pig tracheal chain	Hosseinzadeh and Ghenaati (2006)
Aqueous extract	Stimulatory effect on β2-adrenoreceptors		Nemati et al. (2008)
	Antagonistic effect on muscarinic receptors on smooth muscle of tracheal chain		Neamati and Boskabady (2010)
	Inhibitory effect on histamine H1 receptors		Boskabady et al. (2010)
	Reduced the mean arterial blood pressure and heart rate in a dose‐dependent manner, reductions in mean systolic blood pressure (MABP)	Normotensive and hypertensive rat model	Imenshahidi et al. (2010)
Aqueous extract, Ethanolic extract	Reduced blood pressure in a dose-dependent manner	Isolated rat vas deferens	Fatehi et al. (2003)
Aqueous extract	Antihypertensive effects	Hypertensive rat model	Imenshahidi et al. (2013)
Ethanolic extract	Inhibitory effect on heart rate and contractility	Heart rate and contractility in rat	Boskabady et al. (2008)
Aqueous extract	Induced relaxation via effect on endothelium via nitric oxide synthase and the effect on vascular smooth muscle cells via L type voltage dependent calcium channels	Isolated rat aortic tissue	Razavi et al. (2018)

#### The relaxant effect of kaempferol on smooth muscle

It was demonstrated that flavonoids such as kaempferol have a vasodilatory effect in rat aortic smooth muscle. The results of this *in vitro* study indicated that kaempferol and other flavonoids have a vasodilatory effect via inhibition of PKC that probably is the main vasodilatory mechanism of flavonoids. Flavonoids inhibited cyclic nucleotide phosphodiesterases or decreased Ca^2+^ uptake, which seems to contribute to their vasodilatory effect.

Furthermore, results of this study demonstrated that kaempferol and quercetin were the most potent flavonols against constrictor responses of vasoconstrictors used in this study (PMA, NE and KCL) (Duarte et al. 1993).

*In vitro* administration of flavonoids: genisten (1–100 µM), kaempferol (3–60 µM) and quercetin (1–100 µM) in rat uterus incubated in medium in the presence of different inhibitors proposed that cyclic adenosine monophosphate (cAMP) contributed to the relaxant effect of quercetin and kaempferol on induced tonic contraction by KCL. Furthermore, polyamins contributed to the relaxant effects of kaempferol on KCL-induced tonic contraction but not on CaCl_2_-induced contraction in the depolarised uterus. The other results of this study also indicated that flavonoids have a calcium antagonist action (Revuelta et al. 1997).

It was also shown that kaempferol (3–60 µM) through cAMP produces transcriptional events and polyamines have a relaxant effect on KCL induced tonic contraction in isolated rat uterus (Revuelta et al. 2000). The results of another study about involvement of cAMP in the relaxing effect of flavonoids on rat uterine smooth muscle demonstrated that kaempferol resulted in relaxation of uterine smooth muscle by increasing intracellular cAMP (Revuelta et al. 1999).

The administration of kaempferol (3–60 μM) on KCl (60 mM)-induced contraction in isolated rat uterus showed relaxant effect in a dose-dependent manner. This relaxing effect was antagonised by cAMP-dependent protein kinase inhibitors (Rp-cAMPS and TPCK) and adenylyl cyclase inhibitor (2′,3′-dideoxyadenosine: DDA) (Revuelta et al. 2000).

Butanol extract from soy leaves causes endothelium-independent relaxation in rat carotid artery rings. But, this endothelium-independent relaxation was not observed by six purified kaempferol glycosides from soy leaves (accounting for 48% of the extract in weight). Thus, results suggested that kaempferol glycosides are not responsible for the extract induced relaxation (Ho et al. 2002).

The vasodilatory effect of *in vitro* administration of kaempferol (10^−8^ −10^−4.5^ M) on isolated pulmonary artery of rats was investigated. Experiments were done using the isolated organ bath system by recording tension with the help of data acquisition system, Power lab. Results of this study demonstrated that kaempferol caused concentration-dependent relaxation of endothelium intact pulmonary artery. In endothelium-denuded arterial rings, relaxation produced by kaempferol was not different from the intact artery. L-NAME (NOS inhibitor), indomethacin had not any effect on kaempferol induced relaxation. Furthermore, the administration of other drugs together with kaempferol was done. The results suggested that kaempferol relaxes rat pulmonary artery in endothelium-independent manner through involvement of BKca channel, sGC, PKA pathways and inhibition of Ca^2+^-influx through l-type calcium channels (Mahobiya et al. 2018).

**Figure 1.**
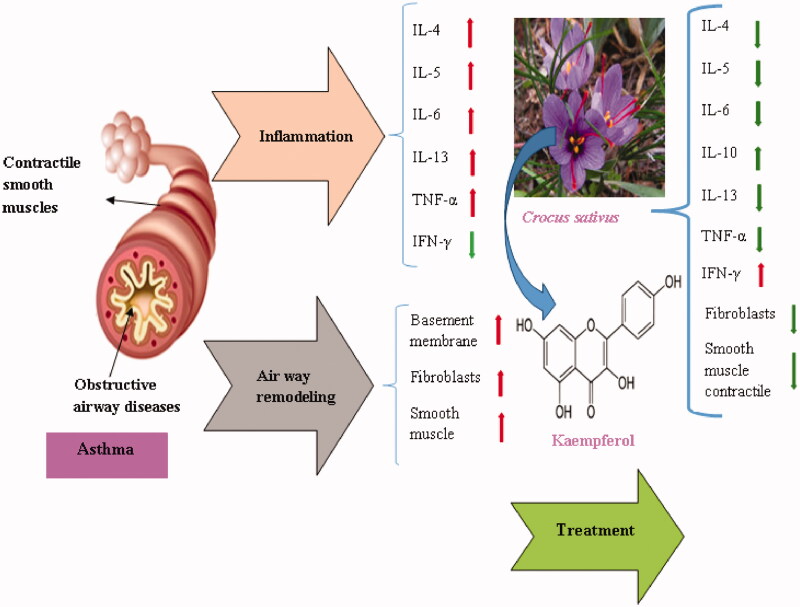
Therapeutic effects of *C. sativus* and kaempferol on inflammatory respiratory diseases.

*In vitro* administration of kaempferol in isolated porcine coronary artery rings were investigated. Contraction-relaxation curve of kaempferol (1 nM–100 µM) was constructed. Kaempferol at high concentration has significant relaxation but this effect was not shown at low concentration. At low concentration (10 µM), kaempferol enhanced relaxation produced by isoproternole, bradykinin, sodium nitroprusside and calcium ionophore A23187 in endothelium intact arteries. In endothelium disrupt rings, kaempferol (10 µM) enhanced relaxation caused by isoproternole, sodium nitroprusside and nifidipine. According to these results, kaempferol at low concentration (10 µM) has no significant vascular effect, but it can enhance endothelium-dependent and endothelium-independent relaxations (Xu et al. 2006). The relaxant effects of kaempferol are shown in Table 4. The therapeutic effects of *C. sativus* and its constituent (kaempferol) on inflammatory respiratory diseases is shown in Figure 1.

**Table 4.** The relaxant effect of kaempferol on smooth muscle.

Effect	Experimental model	Ref.
Showed vasodilatory effect by inhibition of PKC, and cyclic nucleotide phosphodiesterases or decreased Ca^2+^ uptake	Rat aortic smooth muscle	Duarte et al. (1993)
Produced cAMP, and antagonist property on calcium chanel	Rat uterus incubated in medium	Revuelta et al. (1997)
Produces transcriptional events and polyamines through cAMP	Isolated rat uterus	Revuelta et al. (2000)
Increased intracellular cAMP	Rat uterine smooth muscle	Revuelta et al. (1999)
Showed relaxes rat pulmonary artery through involvement of BKca channel, sGC, PKA pathways and inhibition of Ca^2+^-influx via L-type calcium channels	Isolated pulmonary artery of rats	Mahobiya et al. (2018)
Enhanced endothelium – dependent and endothelium – independent relaxations	isolated porcine coronary artery rings	Xu et al. (2006)

## Conclusion

There are two types of common respiratory disorders, including asthma and COPD, which is characterized by airway inflammation, generates excess mucus and smooth muscle dysfunction, making it hard to breathe. Saffron and kaempferol reduced the production of NO, and inflammatory mediators including; IL-4, IL-1β, TNF-α, and MMP-9 in the serum and broncho-alveolar lavage fluid. This plant and its active constituent also increased anti-inflammatory mediators including IFN-γ and IL-10 in *in vivo* and *in vitro* studies. Saffron and kaempferol showed smooth muscle relaxant effects on tracheal smooth muscle (*in vitro* studies) by stimulatory and inhibitory effects on β-adrenoceptor and muscarinic receptors, respectively. The results of this review article indicate *C. sativus* (saffron) and its constituent (kaempferol) have potent therapeutic effects that may be useful for attenuating inflammation in some respiratory diseases such as asthma and COPD.
